# Corneal endothelial cell changes and surgical results after Ahmed glaucoma valve implantation: ciliary sulcus versus anterior chamber tube placement

**DOI:** 10.1038/s41598-021-92420-8

**Published:** 2021-06-21

**Authors:** Joo Yeon Kim, Jihei Sara Lee, Taekjune Lee, Duri Seo, Wungrak Choi, Hyoung Won Bae, Chan Yun Kim

**Affiliations:** Institute of Vision Research, Department of Ophthalmology, Severance Hospital, Yonsei University College of Medicine, 50 Yonsei-ro, Seodaemun-gu, Seoul, 03722 Republic of Korea

**Keywords:** Eye diseases, Glaucoma, Outcomes research

## Abstract

We compared the changes in corneal endothelial cells and surgical outcomes after Ahmed glaucoma valve (AGV) implantation with the valve tip inserted either into ciliary sulcus (CS) or anterior chamber (AC). We retrospectively reviewed the medical records of patients treated with CS AGV (n = 24) and AC AGV (n = 38). We compared the preoperative and postoperative central corneal endothelial cell density (ECD), endothelial cell coefficient of variation (CV), best-corrected visual acuity, intraocular pressure (IOP), number of glaucoma medications, and postoperative complications in the two groups. Both groups had similar baseline characteristics and follow-up period. At the last follow-up, the AC AGV group had significantly higher mean monthly ECD loss (17.47 ± 11.50 cells/mm^2^ vs. 6.40 ± 7.69 cells/mm^2^, *p* < 0.0001) and greater proportion of mean monthly ECD loss than the CS AGV group (0.84 ± 0.53 vs. 0.36 ± 0.39%, *p* < 0.0001). Both groups had similar mean monthly CV changes. The qualified success rates at 2 years were 83.3% and 76.3% for the CS AGV and AC AGV groups, respectively. Although similar surgical outcomes including visual acuity, IOP, number of glaucoma medications, and postoperative complications were obtained following CS AGV and AC AGV, corneal ECD loss was higher in the AC AGV group. Thus, CS AGV may be a better surgical option than AC AGV.

## Introduction

Glaucoma drainage devices (GDD) are commonly used to manage complex and refractory glaucoma. The results of the Tube Versus Trabeculectomy Study revealed that tube shunt surgery had a higher success rate than that of trabeculectomy with mitomycin C during 5 years of follow-up, with similar reductions in intraocular pressure (IOP) and the need for supplemental glaucoma medications^1^. GDD implantation is performed with increasing frequency in high-risk patients in whom trabeculectomy may fail^2,3^.

One of the major complications after GDD implantation, which has recently attracted the attention of many surgeons, is corneal endothelial damage that can lead to subsequent corneal decompensation^4–7^. Considerable efforts have been made to minimize corneal endothelial damage after GDD implantation. In the case of inserting the tube in the anterior chamber (AC), the surgeon tries to shorten the length of the tube and place it as parallel and close to the iris as possible. Nevertheless, there is a possibility of progressive corneal endothelial damage when the tube is located in the AC. Instead of using the conventional method of placing the tube into the AC, the tube may alternatively placed in the pars plana (PP) or ciliary sulcus (CS)^8–15^. However, the placement of the tube in the PP requires vitrectomy, which may lead to additional posterior segment complications or vitreous-associated tube complications. The tube placement in the CS has recently attracted attention, especially in cases of non-vitrectomized pseudophakic eyes. However, only a limited number of studies so far have compared endothelial cell reduction after AC Ahmed glaucoma valve (AGV) and CS AGV implantation^15^. Furthermore, as these studies included combined AGV and cataract surgery it is difficult to assess the effect of AGV-only surgery on corneal endothelial cells.

The aim of this study was to compare the effects of placing the tube in the CS or AC during AGV implantation on corneal endothelial cell loss and surgical outcomes.

## Results

A total of 62 eyes of 62 patients were included, of which 24 were in the CS AGV group and 38 in the AC AGV group. There was no significant difference between the groups in terms of age, sex, laterality, central corneal thickness (CCT), axial length, AC depth, best-corrected visual acuity (BCVA), IOP, number of preoperative glaucoma medications, or type of glaucoma diagnosis (Table 1). The CS AGV group had a significantly higher number of previous intraocular surgeries (1.67 ± 0.64) than the AC AGV group (1.32 ± 0.53, *p* = 0.021). All patients had undergone cataract surgery. The mean time (months) to the last follow-up was slightly longer in the AC AGV (30.45 ± 12.50) than the CS AGV group (24.42 ± 9.88), but this was not statistically significant (*p* = 0.063).

**Table 1:** Preoperative demographics for ciliary sulcus Ahmed valve and anterior chamber Ahmed valve groups.

	CS AGV 24 patients (24 eyes)	AC AGV 38 patients (38 eyes)	*p* value
Age at surgery (years)	65.04 ± 14.53	70.39 ± 11.43	0.129*
Sex, male [n (%)]	17 (70.8)	22 (57.9)	0.449†
Eye, right [n (%)]	13 (54.2)	15 (39.5)	0.384†
**Previous intraocular surgery (n)**	1.67 ± 0.64	1.32 ± 0.53	0.021§
Filtering surgery [n (%)]	7 (29.2)	6 (15.8)	0.347†
Pars plana vitrectomy [n (%)]	6 (17.7)	5 (13.2)	0.702†
CCT (μm)	542.42 ± 34.46	547.76 ± 33.38	0.439*
Axial length (mm)	26.16 ± 3.68	25.07 ± 1.80	0.534*
Anterior chamber depth (mm)	4.56 ± 0.62	4.37 ± 0.62	0.190*
Preoperative BCVA in LogMAR	0.69 ± 0.67	0.47 ± 0.54	0.109*
Preoperative IOP (mmHg)	28.37 ± 6.57	26.00 ± 5.68	0.099*
Preoperative number of glaucoma medications (n)	3.83 ± 0.38	3.79 ± 0.47	0.827*
Follow up period (months)	24.42 ± 9.88	30.45 ± 12.50	0.063*
**Glaucoma diagnosis [n (%)]**			0.466‡
POAG	8 (33.3)	18 (47.4)	
Uveitis	5 (20.8)	4 (10.5)	
NVG	3 (12.5)	6 (15.8)	
Pseudoexfoliation	3 (12.5)	1 (2.6)	
CACG	1 (4.2)	4 (10.5)	
Others^a^	4 (16.7)	5 (13.2)	

*CS AGV* ciliary sulcus Ahmed glaucoma valve, *AC AGV* anterior chamber Ahmed glaucoma valve, *CCT* central corneal thickness, *BCVA* best corrected visual acuity, IOP intraocular pressure, *POAG* primary open angle glaucoma, *NVG* neovascular glaucoma, *CACG* chronic angle closure glaucoma.

*Mann–Whitney test; †χ2 test; ‡Fisher’s exact test.

^§^*p* < 0.05.

^a^Other glaucoma diagnoses include angle recession glaucoma, steroid-induced glaucoma, mixed-mechanism glaucoma, and lens-induced glaucoma.

Values are presented as the mean ± standard deviation.

With regards to preoperative and postoperative IOP and the number of glaucoma medications, comparable results were observed in the two groups throughout the entire follow-up period (*p* > 0.05 at all points, Table 2). At the last follow-up, the mean IOP had significantly decreased from 28.37 ± 6.57 mmHg to 13.42 ± 3.19 mmHg in the CS AGV group (*p* < 0.0001) and from 26.00 ± 5.68 mmHg to 13.26 ± 2.51 mmHg in the AC AGV group (*p* < 0.0001). The mean number of glaucoma medications significantly decreased from 3.83 ± 0.38 to 2.29 ± 1.40 in the CS AGV group (*p* < 0.0001) and from 3.79 ± 0.47 to 2.29 ± 1.35 in the AC AGV group (*p* < 0.0001). The preoperative BCVA values in LogMAR were 0.69 ± 0.67 and 0.47 ± 0.54 in the CS AGV and AC AGV groups, respectively (*p* = 0.109, Table 1). The postoperative BCVA values in LogMAR were 0.77 ± 0.85 and 0.57 ± 0.74 in the CS AGV and AC AGV groups, respectively (*p* = 0.177). There was no significant difference between the preoperative and postoperative BCVA values for both groups (*p* = 0.477 for CS AGV and *p* = 0.127 for AC AGV).

**Table 2:** Preoperative and postoperative intraocular pressure and number of glaucoma medications after ciliary sulcus and anterior chamber Ahmed glaucoma valve procedures.

	IOP (mmHg, mean ± SD)			Number of glaucoma medications (mean ± SD)		
	CS AGV	AC AGV	*p* value*	CS AGV	AC AGV	*p* value*
Preoperative	28.37 ± 6.57	26.00 ± 5.68	0.099	3.83 ± 0.38	3.79 ± 0.47	0.827
**Postoperative**						
POD_1D (CS AGV n = 24 vs. AC AGV n = 38)	8.58 ± 6.72	7.95 ± 5.65	0.821	0.00 ± 0.00	0.00 ± 0.00	1.000
POD_1W (n = 24 vs. n = 38)	8.75 ± 3.98	8.19 ± 3.41	0.495	0.63 ± 0.92	0.47 ± 0.69	0.772
POD_1M (n = 24 vs. n = 38)	16.33 ± 6.17	17.97 ± 5.28	0.270	0.75 ± 1.03	0.58 ± 0.89	0.651
POD_3M (n = 24 vs. n = 38)	14.67 ± 3.21	14.37 ± 3.04	0.805	1.71 ± 1.60	1.66 ± 1.40	0.899
POD_6M (n = 24 vs. n = 38)	13.33 ± 2.33	14.42 ± 2.15	0.079	1.87 ± 1.45	1.92 ± 1.42	0.970
POD_1Y (n = 24 vs. n = 38)	13.71 ± 3.06	13.95 ± 2.75	0.566	1.96 ± 1.52	2.03 ± 1.37	0.888
POD_1.5Y^a^ (n = 19 vs. n = 33)	14.00 ± 2.19	14.27 ± 2.75	0.491	2.00 ± 1.49	2.21 ± 1.36	0.660
POD_2Y^b^ (n = 13 vs. n = 28)	13.62 ± 1.61	13.93 ± 2.74	0.661	1.92 ± 1.50	2.36 ± 1.47	0.359
Final visit (n = 24 vs. n = 38)	13.42 ± 3.19	13.26 ± 2.51	0.884	2.29 ± 1.40	2.29 ± 1.35	0.959

*SD* standard deviation, *CS AGV* ciliary sulcus Ahmed glaucoma valve, *AC AGV* anterior chamber Ahmed glaucoma valve, *IOP* intraocular pressure, *POD* postoperative day, *D* day, *W* week, *M* month, *Y* year.

*Mann–Whitney test.

^a^Number of patients for csAGV and acAGV are 19 and 33, respectively.

^b^Number of patients for csAGV and acAGV are 13 and 28, respectively.

For those not mentioned, 24 csAGV and 38 acAGV cases were compared.

Values are presented as the mean ± standard deviation.

**Figure 1:**
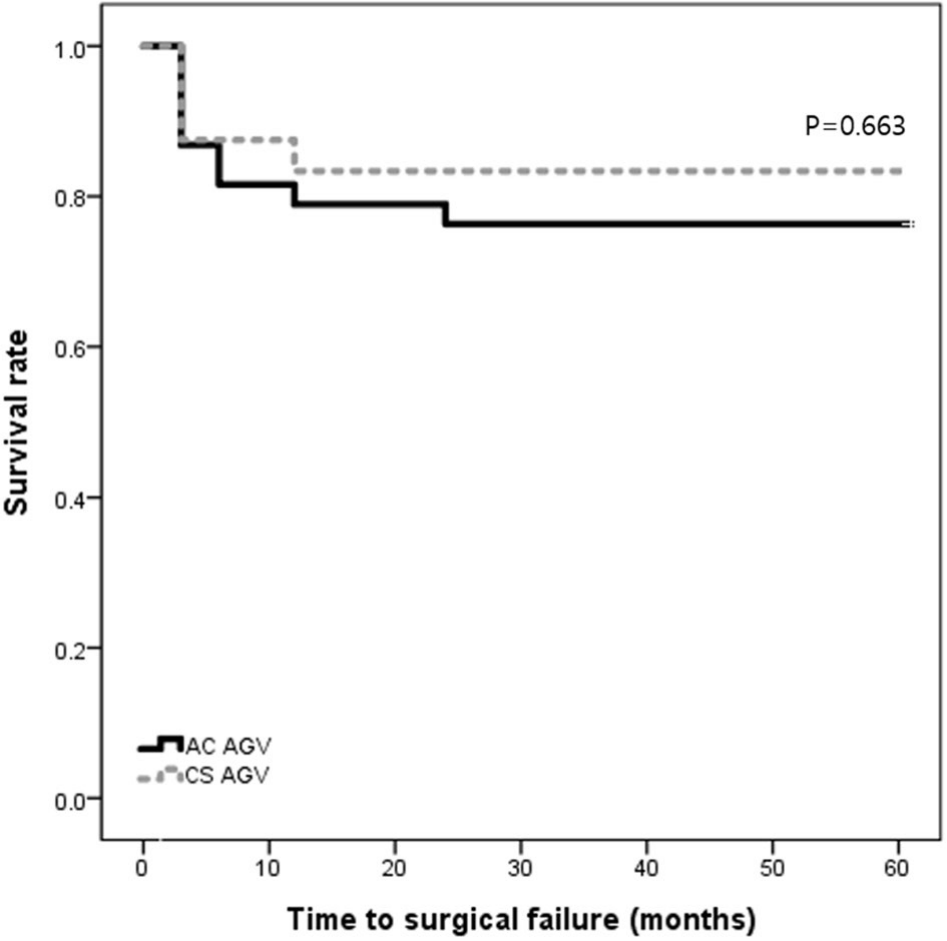
Kaplan–Meier survival curves after ciliary sulcus and anterior chamber Ahmed glaucoma valve implantation. The success rates were 83.3% and 78.9% at 12 months, and 83.3% and 76.3% at 24 months in the ciliary sulcus and anterior chamber groups, respectively. The difference between the survival curves of the two groups was not statistically significant (*p* = 0.663 by Log-rank test).

Figure 1 shows the Kaplan–Meier survival analysis of surgical success for both groups. The success rates were 83.3% and 78.9% at 12 months, and 83.3% and 76.3% at 24 months in the CS AGV and AC AGV groups, respectively. The number of eyes included for survival analysis were 12 and 26 at 12 months, and 10 and 14 at 24 months in the CS AGV and AC AGV groups, respectively. The difference between the survival curves was not statistically significant (*p* = 0.663 by Log-rank test). In the CS AGV group, one patient required an additional glaucoma surgery (secondary CS AGV) at 12 months, while another patient, whose preoperative BCVA was hand motion with uncontrolled neovascular glaucoma, lost light perception. Five patients were considered surgical failure due to inadequate IOP reduction. Three failed at 3 months post-op and the other two failed at 1 year post-op. The surgical failure for the AC AGV group included two patients who required removal of the device due to low ECD count (postoperative ECD 756 and 588 cells/mm^2^ with ECD loss of 788 and 205 cells/mm^2^, respectively) and one patient with primary open angle glaucoma (POAG), who lost light perception from preoperative visual acuity of hand motion. The rest of patients were considered surgical failure because they no longer met the 20% reduction criterion; 5 showed inadequate IOP reduction at 3 months post-op, 2 at 6 months post-op, 1 at 1 year post-op and another at 3 years post-op.

Postoperative complications are summarized in Table 3. Some manageable postoperative complications without any statistically significant differences were observed between the groups. In both groups, the most common complication was transient hypotony, defined as IO* p* ≤ 5 mmHg, which was observed in six eyes (25.0%) in the CS AGV group and eight eyes (21.1%) in the AC AGV group (*p* = 0.717). Hyphema occurred slightly more often in the CS AGV group than in the AC AVG group (CS AGV five eyes [20.8%] vs AC AGV three eyes [7.9%]), but the difference was not statistically significant (*p* = 0.242). The CS AGV group had two cases of vitreous hemorrhage. In all cases, hyphema and vitreous hemorrhage resolved without the need for additional procedures during the follow-up period. Both groups had one case of choroidal detachment, which resolved spontaneously during follow-up. In the AC AGV group, one case of flat anterior chamber required viscoelastic injection into the anterior chamber. There was one case of an intraocular lens dislocation that required operation in the AC AGV group. However, in that case, the capsular bag had already been unstable even before the operation, hence, it was not considered to be related to AC AGV. In the CS AGV group, two eyes showed anterior tenting of the iris as the tube was pushing the iris forward. Postoperatively, in one eye with iris tenting, the iris-cornea angle decreased from 41.7° to 21.9° and the iris-cornea distance decreased from 1.91 mm to 1.09 mm (Fig. 3A–C). In the other case with iris tenting, the changes in the iris-cornea angle and iris-cornea distance were mild (Fig. 3D–E). Both cases with iris tenting exhibited comparable monthly ECD loss (3.23 cells/mm^2^ and 4.16 cells/mm^2^, respectively) when compared to the monthly ECD loss of the CS AGV group (6.40 ± 7.69 cells/mm^2^). In most CS AGV cases, there were no specific comments in the medical records about iris tenting since it was considered mild.

**Table 3:** Postoperative complications after ciliary sulcus and anterior chamber Ahmed glaucoma valve procedures.

Complication	CS AGV^a^ (24 eyes)	AC AGV^a^ (38 eyes)	*p* value
Transient hypotony^b^	6 (25.0)	8 (21.1)	0.717*
Hyphema	5 (20.8)	3 (7.9)	0.242†
Vitreous hemorrhage	2 (8.3)	0 (0)	0.146†
Choroidal detachment	1 (4.2)	1 (2.6)	1.000†
Flat anterior chamber^c^	0 (0)	1 (2.6)	1.000†
IOL-related complication	0 (0)	1 (2.6)	1.000†

Values are presented as the number of cases (percentage).

*CS AGV* ciliary sulcus Ahmed glaucoma valve, *AC AGV* anterior chamber Ahmed glaucoma valve, *IOL* intraocular lens.

*χ^2^ test; †Fisher’s exact test.

^a^Data presented as number of patients (percentage).

^b^Defined as intraocular pressure ≤ 5 mmHg.

^c^Requiring viscoelastic injection into the anterior chamber.

**Figure 2:**
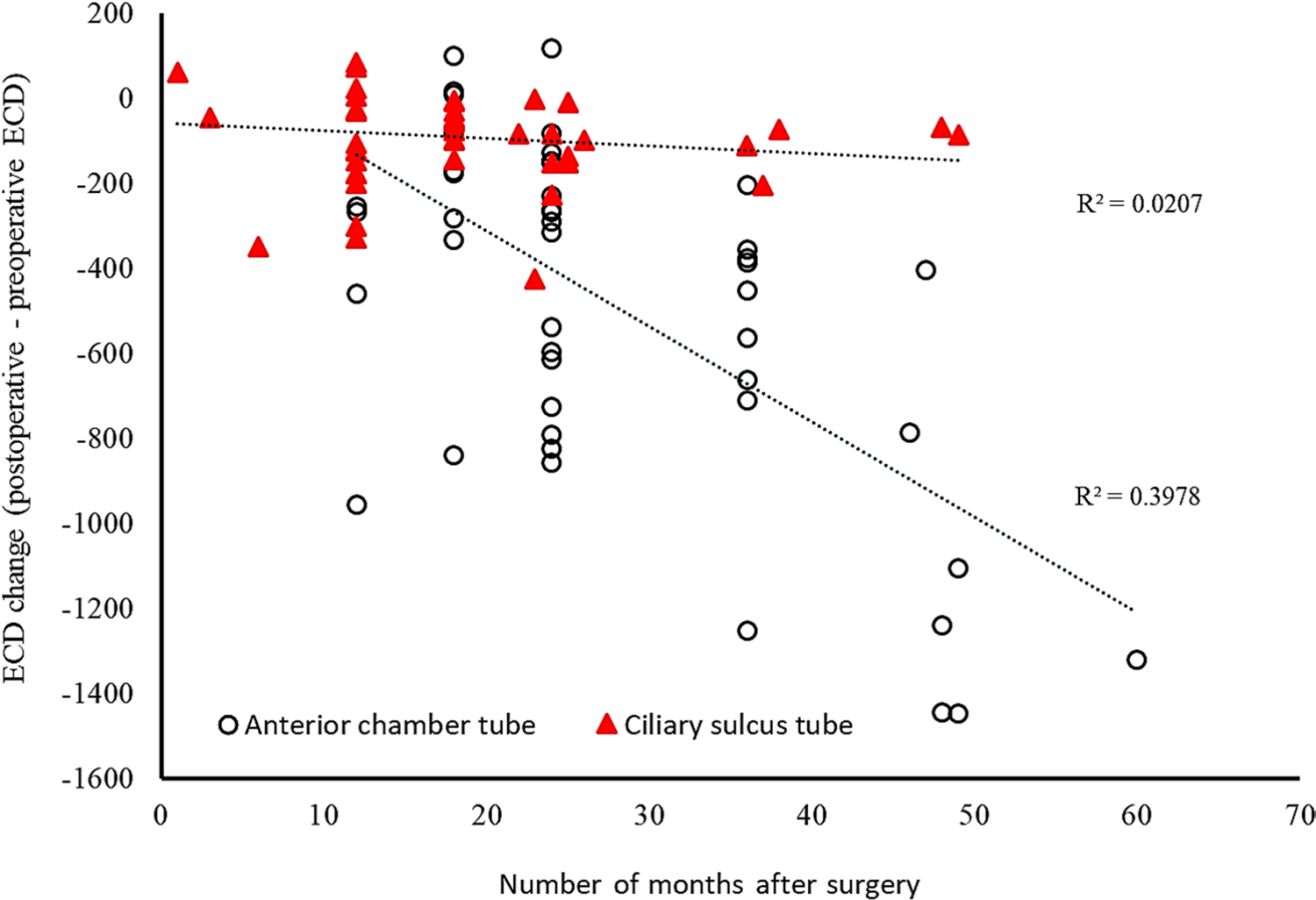
Scatterplots of ECD loss over time following Ahmed glaucoma valve implantation. The tubes were placed either in the anterior chamber (circle) or in the ciliary sulcus (red arrowhead).The horizontal axis shows the time elapsed from the time of surgery to the repeat ECD measurement in months. The vertical axis shows ECD change between preoperative and postoperative measurements. The goodness of the fit to the regression line is shown as r^2^.

The preoperative and postoperative endothelial cell measurements in both groups are summarized in Table 4. Both groups showed similar preoperative ECD (CS AGV 1918.42 ± 653.90 vs AC AGV 2107.37 ± 522.36 cells/mm^2^, *p* = 0.406). Postoperative ECD significantly decreased in both groups compared to baseline ECD (1799.21 ± 662.29 cells/mm^2^, *p* < 0.0001 in the CS AGV group and 1587.39 ± 611.73 cells/mm^2^, *p* < 0.0001 in the AC AGV group). At the last follow up, ECD loss (defined as preoperative ECD – postoperative ECD) in the AC AGV group was 519.97 ± 385.01 cells/mm^2^, which was significantly greater than that in the CS AGV group (119.21 ± 105.93 cells/mm^2^, *p* < 0.0001), and the percentage of ECD loss (defined as ECD loss/preoperative ECD × 100) also showed the same trend (AC AGV 25.60 ± 18.54% vs CS AGV 7.12 ± 7.78%, *p* < 0.0001). The mean monthly ECD loss was calculated by dividing the ECD loss by the number of follow-up months since the surgery. The mean monthly ECD loss was significantly higher in the AC AGV group (17.47 ± 11.50 cells/mm^2^) than that of the CS AGV group (6.40 ± 7.69 cells/mm^2^, *p* < 0.0001). The monthly percentage of ECD loss, calculated by dividing the mean monthly ECD loss by the preoperative ECD, showed greater change in the AC AGV than the CS AGV group (0.84 ± 0.53% vs 0.36 ± 0.39%, *p* < 0.0001). The mixed effects model showed that the mean decrease in ECD at 24 months was 19.60 ± 2.81% for the AC AGV group and 9.97 ± 3.84% for the CS AGV group at 24 months (*p* = 0.049) and 30.31 ± 3.66% for the AC AGV group and 7.11 ± 7.16% for the CS AGV group at 36 months (*p* = 0.006). When age at operation and number of previous ocular operations were taken into account, the mean decrease at 36 months was 30.42 ± 3.67% and 7.73 ± 7.19% for the AC AGV and CS AGV groups, respectively (*p* = 0.008). In order to account for the differences in the time lapse since the surgery, mean monthly changes were calculated and scatterplots were drawn to compare the ECD change over time between the two groups (Fig. 2). Both the preoperative and monthly CV changes did not show significant differences between the two groups.

**Table 4:** Comparison of corneal endothelial cell measurements between the ciliary sulcus Ahmed valve and anterior chamber Ahmed valve groups.

	CS AGV 24patients (24 eyes)	AC AGV 38 patients (38 eyes)	*p* value*
**ECD**			
Preoperative ECD (cells/mm^2^)	1918.42 ± 653.90	2107.37 ± 522.36	0.406
ECD loss (cells/mm^2^)^a^	119.21 ± 105.93	519.97 ± 385.01	< 0.0001†
% ECD loss (%)^b^	7.12 ± 7.78	25.60 ± 18.54	< 0.0001†
Monthly ECD loss (cells/mm^2^)^c^	6.40 ± 7.69	17.47 ± 11.50	< 0.0001†
% Monthly ECD loss (%)^d^	0.36 ± 0.39	0.84 ± 0.53	< 0.0001†
**CV**			
Preoperative CV	40.13 ± 8.49	37.61 ± 10.78	0.183
Monthly CV change^e^	− 0.10 ± 0.39	− 0.15 ± 0.35	0.394

*CS AGV* ciliary sulcus Ahmed glaucoma valve, *AC AGV* anterior chamber Ahmed glaucoma valve, *ECD* Endothelial cell density.

*Mann–Whitney test.

^†^*p* < 0.05.

^a^ECD loss = Preoperative ECD – Postoperative ECD.

^b^% ECD loss = (Preoperative ECD – Postoperative ECD) / preoperative ECD × 100.

^c^Monthly ECD loss = monthly ECD loss.

^d^% Monthly ECD loss = monthly ECD loss / preoperative ECD × 100.

^e^Monthly CV change = monthly CV change.

Values are presented as the mean ± standard deviation.

Table 5 shows the results of univariate and multivariate analyses performed to assess clinical variables associated with monthly ECD loss after the AGV implantation procedure in both groups. In univariate analyses, more preoperative ECD (*p* = 0.032), fewer previous intraocular surgeries (*p* = 0.010), and placement of AGV tube tip in the AC (*p* < 0.0001) were significantly associated with greater monthly ECD loss. In multivariate analyses, only tube location in the AC (*p* = 0.001) showed significant association with greater monthly ECD loss.

**Table 5:** Results of univariate and multivariate analysis of the clinical variables associated with monthly endothelial cell density loss^a^ after Ahmed glaucoma valve implantation (N = 62 eyes, 62 patients).

	Univariate analysis			Multivariate analysis		
	Estimate	Standard error	*p* value	Estimate	Standard error	*p* value
Age	− 0.046	0.115	0.692			
Male sex (vs. female sex)	3.111	3.021	0.307			
Uveitis diagnosis (vs. others)	− 3.480	4.154	0.405			
Preoperative ECD	0.005	0.002	0.032*	0.003	0.002	0.225
Preoperative IOP	0.149	0.243	0.541			
Postoperative IOP	0.448	0.533	0.404			
Follow up period in months	− 0.017	0.125	0.896			
Number of previous intraocular surgeries	− 6.293	2.373	0.010*	− 2.825	2.442	0.252
Ciliary sulcus tube location (vs. Anterior chamber)	− 11.072	2.663	< 0.0001*	− 9.520	2.729	0.001*

*ECD* endothelial cell density, *IOP* intraocular pressure.

*p < 0.05.

^a^ECD loss = Preoperative ECD – Postoperative ECD.

## Discussion

In this study, we compared the difference in ECD loss and surgical outcomes associated with the AGV tube insertion position, in the AC or CS. We found that AGV implantation in the CS was associated with less ECD loss compared to that in the AC with comparable surgical results.

ECD loss after AC AGV insertion has been addressed by several studies^5,17^. After AC AGV insertion, an ECD loss of 10.5–12.6% was reported at 12 months^4,5,17^, 14.1% at 18 months^5^, and 15.4–16.9% at 24 months^4,5^. Although several hypotheses have been proposed to explain the mechanism of ECD loss after AGV surgery, such as turbulent flow at the tip of the implant, postoperative inflammation, depletion of nutrients and oxygen, intermittent tube-corneal/uveal touch, and foreign body effect by silicone tube, the exact underlying mechanism remains unclear^18,19^. Inserting the tube tip in the PP or CS instead of the AC has been attempted to overcome the ECD loss^9,14,15,20^. One study that compared the ECD change between PP AGV and AC AGV, reported that at 18 months, PP AGV showed less average percentage decrease of central ECD (12.5%) compared to that in AC AGV (18.4%)^9^. Murakami et al., reported that at 24 months after inserting the Ahmed and Baerveldt drainage device in the CS, ECD decreased by 14.6 ± 5.4%^20^. They also reported that a decrease in ECD was associated with an additional preoperative intraocular surgery and higher preoperative IOP, and not with the type of drainage device used. Zhang et al., reported that the mean monthly loss of central ECD was significantly higher in the AC AGV group (29.3 ± 29.7 cells/mm^2^) than the CS AGV group (15.3 ± 20.7 cells/mm^2^), including eyes that had undergone concurrent phacoemulsification and AGV^15^.

Our results were similar to earlier studies in that the ECD loss was less in the CS AGV group than in the AC AGV group^15^. By placing the drainage tube into the CS instead of AC, the distance between the tube and the corneal endothelium is increased and the effects from the turbulent flow at the tip of the implant or intermittent tube-corneal touch are mitigated. The iris may also act as a barrier to mechanical factors that cause ECD loss. The degrees of monthly ECD loss in this study was relatively low in both groups (7.12 ± 7.78 in the CS AGV group and 25.60 ± 18.54 cells/mm^2^ in the AC AGV group). Especially, the ECD loss rate was especially low for the CS AGV group. One possible reason for the discrepancy in the results between our study and previous published studies is that patients with concurrent phacoemulsification were not included in this study. The effect of concurrent phacoemulsification on the corneal endothelium through additional inflammation or phacoemulsification energy was excluded. Another possible reason is the relatively shorter period of follow-up for our study. Lower numbers of patients with underlying diseases in comparison may also have contributed to the discrepancy.

Bayer and Önol retrospectively compared the clinical outcomes of 68 eyes with AC AGV and 35 eyes with CS AGV, and found no difference in IOP control and the number of postoperative glaucoma medication between the CS AGV and AC AGV groups^12^. They also reported that the incidence of flat AC was higher in the AC AGV group. Zhang et al., reported that the mean IOP did not differ between the AC AGV and CS AGV groups at the last follow-up (37.6 months for the AC AGV group and 20.1 months for the CS AGV group), but BCVA was significantly worse in the AC AGV group compared to that of the CS AGV group^15^. In this study, clinical outcomes were comparable between the two groups, including postoperative IOP control, the number of medication, BCVA, and postoperative complications. The success rates at 24 months were 83.3% and 76.3% in the CS AGV and AC AGV groups, respectively, which were similar to previously reported success rates after AC AGV (ranging from 73.6% to 84.2%, with mean follow-up ranging from 12 to 24 months)^21–25^.

In this study, postoperative hyphema was observed in 20.8% and 7.9% of cases after CS AGV and AC AGV, respectively. As the CS is more vascular structure than the AC angle, transient hyphema may occur more frequently after CS AGV. Postoperative hemorrhage, such as hyphema or vitreous hemorrhage, resolved without additional interventions. There was a case of intraocular lens (IOL) dislocation in the AC AGV group, which required surgical intervention. However, since instability of the capsular bag had been observed in that case before the surgery, it was not considered a result of the surgical method. Although IOL-related problems, such as IOL dislocation or obstruction of the tube by an IOL, were not observed in the CS AGV group, preoperative findings, including zonular weakness, should be carefully examined before placing the tube in the CS. Placing the tube in the CS may cause iris tenting due to the tube tip pushing the iris forward^15^, thereby decreasing the distance between the tube and the cornea. Although postoperative anterior segment-optical coherence tomography (AS-OCT) analysis was not performed for all CS AGV patients, iris tenting seemed not to affect postoperative ECD loss similar to the findings of a previous report^15^. Furthermore, if the tube pushes the iris in the CS AGV, it may worsen or cause intraocular inflammation. However, even when the tube was tenting the iris, there was no sign of any specific intraocular inflammation.

Our study has several limitations. First, due to the retrospective study design, all ECD measurements were not taken during the same interval, which may have affected the observed patterns of change. However, this was compensated to some extent by the fact that ECD was measured four or more times in most patients. Second, although the AC AGV group had a longer follow-up period than the CS AGV group as the surgical method used changed in 2017 (from AC AGV to CS AGV), there was no statistical difference in the follow-up periods of the two groups. In addition, although the degree of damage may vary due to the difference in the follow-up period, this may be corrected to some extent by assessing the changes month wise. Third, we excluded eyes with preoperatively compromised cornea or eyes that had previously undergone keratoplasty or complicated intraocular surgeries. Thus, we could not compare ECD changes or surgical results in vulnerable eyes that may have benefited from this surgical alteration. Lastly, we evaluated only the central area of the corneal endothelium, and thus, did not evaluate ECD changes in other areas. Further prospective studies with annual specular data, not only in the central area but also in the peripheral areas, will provide more insights regarding the pattern of ECD change.

In summary, AGV implantation in the CS was associated with less ECD loss compared with that of AGV implantation in the AC, while the postoperative outcomes were comparable. Thus, AGV implantation in the CS may be preferred to prevent ECD loss.

## Methods

This was a single-center, retrospective comparative case series study approved by the Institutional Review Board of Severance Hospital (No. 2020-3784-001). Written informed consents were waivered due to the retrospective nature of the study. Furthermore, this study followed the tenets of the Declaration of Helsinki. We reviewed the medical records of patients with refractory glaucoma unresponsive to maximal medical treatment who underwent implantation of an AGV (Model FP-7, New World Medical, Inc., Rancho Cucamonga, CA) either in the AC or in the CS between 2013 and 2019. Patients with at least 6 months follow-up period and who had preoperative and postoperative specular microscopic examination taken after at least 6 months of follow-up were included in this study. Exclusion criteria included previous GDD implantation in the same eye, preexisting corneal disease that could affect corneal endothelium, previous corneal transplant, phakic/aphakic status, those with a history of laser refractive surgery, concurrent cataract surgery performed at the same day of AGV procedure, and eyes that underwent any intraocular surgery within one year prior to AGV insertion. If both eyes met the criteria, the first eye operated on was included.

### Surgical techniques

All surgeries were performed by a single surgeon (CYK). The AGV tube tip was traditionally placed in the AC, but since 2017, the surgeon gradually changed the method and started placing the AGV tube tip in the CS. AGV implantation was performed following a previously reported method^16^. Under sub-Tenon anesthesia, a fornix-based conjunctival incision was made and the Tenon capsule was dissected using spring scissors. Two flaps, a 4 × 4 mm right-angled triangular shaped partial thickness scleral flap and a continuous 2 mm-wide × 6 mm-long bridge-shaped partial-thickness scleral flap, were made at the superotemporal quadrant. Tube priming was performed with balanced salt solution irrigation, and the AGV body was placed 8–10 mm posterior to the limbus and between the rectus muscles. In the AC AGV group, the tube tip was cut to an adequate length, in a bevel-up manner. Using a 23-gauge needle, sclerotomy was created 2–3 mm posterior to the limbus under the scleral flap, entering the AC. The tube was inserted into the AC, parallel to the iris plane. In the CS AGV group, the tube tip was trimmed in a bevel-down manner, with a length enough to be seen through a dilated pupil. After pre-filling the CS area with viscoelastics, sclerotomy was performed using a 23-gauge needle, 2–3 mm posterior to the limbus underneath the scleral flap. The needle was advanced into the CS until the tip of the needle could be visualized in the pupillary area, and the tube was inserted along the sclerotomy path. For both groups, the scleral flap was repositioned over the tube and sutured using a 10–0 nylon suture, and the conjunctiva and Tenon’s capsule were then secured at the limbus with interrupted 8–0 vicryl sutures. Topical steroid and antibiotic eye drops were prescribed for 8 weeks, and the patients were followed closely.

### Examinations

By reviewing the medical records, we collected preoperative demographics and ocular characteristics including the patients’ age, sex, glaucoma diagnosis, laterality, IOP, number of glaucoma medications, BCVA in logMAR (minimal angle of resolution), previous intraocular surgery, CCT, axial length, and AC depth. Postoperative data including IOP, and the number of glaucoma medications at day 1, week 1, months 1, 6, 12, and 18, and every year thereafter until the last date of follow-up were obtained. Postoperative surgical complications were assessed at all follow-up periods. Specular microscopic examination was performed by experienced examiners using a non-contact specular microscope (Topcon SP-3000P; Topcon Corp., Tokyo, Japan) within one month prior to each surgery. The central corneal area was imaged while the patient fixed gaze at the target in the instrument. The manual center-dot method was used to measure the central corneal ECD and CV, marking at least 50 contiguous endothelial cells. Specular microscopy was repeated at the last follow-up. If additional intraocular surgery was needed, follow-up was discontinued to exclude possible effects on the corneal endothelium. Eyes meeting the following criteria were considered surgical success: IO* p* > 21 mmHg or less than 20% reduction below baseline after 3 months, no repeat surgery for glaucoma (AGV, cyclodestruction, or adjusting of the valve), no removal of the implant, and no loss of light perception^1^. The need for supplemental medical therapy to lower IOP following surgery was not considered in the definition of surgical success. In the CS AGV group, some eyes received postoperative AS-OCT (Casia SS-1000; Tomey, Nagoya, Japan) to evaluate the presence of iris tenting. If iris tenting was noted, the iris-cornea angle (the angle between the anterior surface of the iris and the posterior surface of the cornea) and the iris-cornea distance (the distance between the most anterior part of tented iris and the cornea) were measured and compared with preoperative measurements.

### Statistical analysis

Continuous variables were presented as mean ± standard deviation and categorical variables were presented as number (percentage). The Shapiro–Wilk test was performed to assess data distribution patterns. Continuous variables were compared using the Mann–Whitney *U* test and categorical variables were compared using the Chi-square test or Fisher’s exact test (for comparisons between two groups). The Wilcoxon signed rank test was performed to compare preoperative and postoperative characteristics. Kaplan–Meier survival analysis and log-rank test were performed to analyze the cumulative probability of success in both groups. Univariate and multivariate regression analyses, using the enter method, were performed to access the variables associated with monthly ECD loss. Results with *p* < 0.05 were considered statistically significant. The mentioned statistical analyses were performed using the SPSS ver. 24.0 (IBM Corp., Armonk, NY). Linear mixed-effects models with patient-specific random intercepts were used to account for differencesin the number and time points of endothelial cell density measurements in assessing the changes in the cell density over time. SAS version 9.4 (SAS Inc., Cary, NC, USA) was used for the analysis.

**Figure 3:**
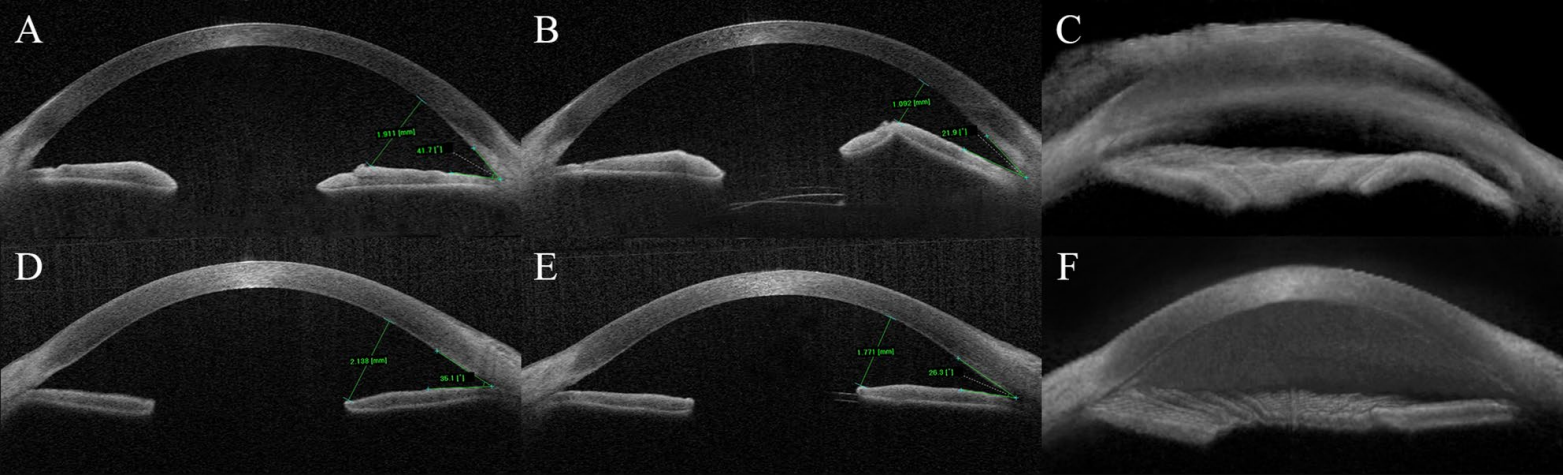
Anterior segment optical coherence tomographic images in two patients with iris tenting after ciliary sulcus Ahmed glaucoma valve implantation. Preoperative (**A**, **D**), postoperative (**B**, **E**), and 3D images (**C**, **F**). In one patient with prominent iris tenting, the iris-cornea angle decreased from 41.7° to 21.9° and the iris-cornea distance decreased from 1.91 mm to 1.09 mm (**A**–**C**). In the other patient with mild iris tenting, the iris-cornea angle decreased from 35.1° to 26.3° and the iris-cornea distance decreased from 2.14 mm to 1.77 mm (**D**–**F**).

## Data Availability

The datasets generated during and/or analyzed during the current study are available from the corresponding author on reasonable requests.
